# Synthesis of silk fibroin and silver nanoparticles for enhanced functionality in cellulosic textiles

**DOI:** 10.1038/s41598-025-22602-1

**Published:** 2025-11-04

**Authors:** M. A. M. Pranto, M. Kabir, M. A. Haque, M. S. Hasan, J. Saha, M. Rahman, T. Islam, S. C. Das

**Affiliations:** 1 Department of Textile Engineering, Mawlana Bhashani Science and Technology University, Tangail, 1902 Bangladesh; 2 College of Textiles and Clothing, Qingdao University, Qingdao, 266071 China; 3 Department of Textile Engineering, Jashore University of Science and Technology, Jashore, 7408 Bangladesh; 4 Department of Textiles, Merchandising, and Interiors, University of Georgia, Athens, GA 30602 USA; 5 Department of Manufacturing and Civil Engineering, Norwegian University of Science and Technology, 2815 Gjøvik, Norway

**Keywords:** Cellulosic textiles, Silk nanoparticles, Silver nanoparticles, Nano-modification, Functional materials, Medical textiles

## Abstract

This study investigates the synthesis of silk fibroin nanoparticles (SFNPs) and silver nanoparticles (AgNPs) and their application to cotton textiles to enhance functional properties for potential biomedical use. The nanoparticles were synthesized using chemical reduction and nanoprecipitation methods, and their formation and stability were confirmed through UV–Vis spectroscopy, X-ray diffraction (XRD), Fourier-transform infrared spectroscopy (FTIR), and scanning electron microscopy with energy-dispersive spectroscopy (SEM–EDS). Cotton fabrics were subsequently modified with SFNPs, AgNPs, and a combined SF-AgNPs formulation. Characterization confirmed the successful deposition and interaction of nanoparticles with cellulose fibers. The treated textiles demonstrated improved antibacterial activity against *Staphylococcus aureus* and *Escherichia coli*, along with enhanced antioxidant performance as evidenced by DPPH radical scavenging assays. Notably, the combined SF-AgNPs treatment exhibited synergistic effects, providing stronger antimicrobial durability and higher antioxidant capacity compared to single-nanoparticle treatments. These findings highlight the potential of SFNPs and AgNPs as effective nanomaterials for producing multifunctional, bioactive cotton textiles with promising applications in healthcare and biomedical fields.

## Introduction

The popularity of cellulosic textiles, particularly cotton textiles, is due to their user-friendly properties, such as softness, hygroscopicity, skin affinity, biodegradability, and regeneration capability^1^. These attributes make them ideal for everyday use. However, the proliferation of microorganisms in cotton textiles will likely impede maintaining hygiene and cleanliness despite these beneficial properties^2^. The demand for antibacterial and functional textiles has increased with growing consumer demand for health and hygiene. Recently, there has been significant interest in using textile materials for biomedical applications such as drug delivery, tissue engineering, or bio-imaging^3,4^. This interest is driven by the exceptional characteristics of these materials. Nanomaterials or nanoparticles (NPs) have attracted considerable attention due to their unique physicochemical characteristics, making them highly sought after in various fields^5,6^. NPs like titanium dioxide (TiO_2_), zinc oxide (ZnO), silicon dioxide (SiO_2_), and silver nanoparticles (AgNPs) have been explored for their potential in textile materials for developing various functional properties^7^. The antimicrobial activities of silver (Ag) have been well documented since ancient times, leading to its widespread use in the pharmaceutical and biomedical fields^8^, particularly noted for their broad-spectrum antibacterial or antimicrobial action^9,10^. They also demonstrated very low cytotoxicity to mammalian cells^11,12^. The nanoparticles (AgNPs) can be synthesized using varied reducing agents such as sodium borohydride, sodium citrate, soluble starch, and polymers polyvinyl pyrrolidone (PVP), polyethylene glycol (PEG), and starch^13^. Surfactants can be employed as stabilizers to avoid nanoparticle agglomeration^14^.

Silk fibroin (SF) is a naturally occurring protein primarily obtained from silk fibers secreted from silkworms^15,16^. The fibrous protein forms the primary framework of silk, contributing to its strength, flexibility, and brilliance^17^. SF consists of two leading polypeptide chains, fibroin heavy chain (Fib–H) and fibroin light chain (Fib–L), bound together via disulfide bonds^18^. The primary source of SF is cocoons formed by silkworm larvae during silk production. These cocoons are processed by removing the outer layer (sericin) and harvesting the fibroin fibers^19,20^. In nanoparticle form, silk fibroin nanoparticles (SFNPs) exhibit several advantages, including biocompatibility, biodegradability, non-toxicity, and high surface functionality. Their nanoscale size provides a large surface area and tunable surface chemistry, making them effective carriers for bioactive molecules and enhancers of fabric performance. SFNPs are reported to possess moisture management, antioxidant activity, UV protection, and mild antimicrobial effects, in addition to providing a stable coating layer on fibers^21^. When applied to cotton textiles, SFNPs improve softness, durability, and surface roughness, while offering active functional sites that facilitate binding with silver nanoparticles (AgNPs) or other additives. This synergistic role not only improves the adhesion and stability of AgNPs on cotton but also enhances the antioxidant, antibacterial, and protective properties of the coated fabrics^22^. The preparation of a composite colloid composed of AgNPs and SF involves using SF as both a reducing and dispersion agent. The experimental process starts with the selective oxidation of cotton textiles using NaIO_4_ to produce oxidized cotton fabrics^23^. These textiles are then treated with the SF-AgNPs hybrid colloid in a one-step process, where SF reduces silver nitrate and disperses AgNPs on the textile surface. The chemical transformation of oxidized cotton textiles through cross-linking with SF results in antibacterial properties. The combination of SF and AgNPs offers benefits over conventional methods^24,25^.

Several studies have explored the employment of silk fibroin (SF) and silver nanoparticles (AgNPs) on cellulosic textiles to enhance their functionality. Among them, Repon et al.^26^ demonstrated that incorporating AgNPs into cellulosic textiles significantly enhances their antibacterial efficacy against the Gram-positive bacterium *S. aureus* and the Gram-negative bacterium *E. coli.* The durability of these materials was also assessed after 10 washes. Overall, very good ratings of color fastness were recorded regarding wash, water, and perspiration, as well as rubbing and light fastness. Another study, Ibrahim et al. prepared AgNPs and applied to a cotton fabric, followed by gamma-radiation or thermal curing. Showing high durability of the attained antimicrobial activities, even after 20 washing cycles; where they achieved 99.1% and 98.7% reduction of *E. coli* and *S. aureus* bacterial counts, respectively^27^. In a similar study, Yue et al.^28^ synthesized silver nanoparticles-sericin (Ag NPS-sericin) hybrid colloid using sericin as both reducing and dispersing agent. When applied to NaIO₄-oxidized cotton fabrics, the treated textiles exhibited strong antibacterial activity, with a bacterial reduction rate of 99.28% for *S. aureus* and 99.06% for *E. coli*, at an initial silver content of 136.72 mg/kg. Wang et al.^29^ synthesized AgNPs using Bacillus methylotrophicus after 48 h of incubation at 28 °C, demonstrating superior growth inhibition against *Candida albicans*, *Escherichia coli*, *Salmonella enterica*, and *Vibrio parahaemolyticus* compared to antibiotics. Similarly, Karunakaran et al.^30^ prepared spherical AgNPs (20–70 nm diameter) from Aztobacter vinelandii extracts, exhibiting high antioxidant and antibacterial activities against pathogens, including *S. aureus, E. coli, S. fradiae*, and *S. marcescens*, with applications in nanomedicine, health, food, and pharmaceuticals^31^.

Previous studies have shown that incorporating silver nanoparticles (AgNPs) onto cellulosic textiles significantly enhances their antibacterial and functional properties. However, there is limited research on combining AgNPs with natural protein-based nanoparticles like silk fibroin nanoparticles (SFNPs), which offer excellent biocompatibility and sustainability. In this study, AgNPs are synthesized using a controlled chemical reduction method, while SFNPs are derived from silk fibroin extracted from *Bombyx mori* cocoons through an optimized degumming and dissolution process. The synthesized nanoparticles are applied to cotton fabrics both individually and in combination (AgNPs + SFNPs) to investigate their synergistic effects on antimicrobial performance and fabric durability. This research aims to fill the gap by exploring a novel approach to develop multifunctional, nano-enhanced textiles with potential applications in biomedical and protective materials. The paper includes detailed methodologies for nanoparticle synthesis, fabric treatment processes, and performance evaluations of the modified cotton fabrics.

## Experimental

### Materials

*Bombyx mori* silk cocoons were collected from the Bangladesh Sericulture Research Training Institute (BSRTI), Rajshahi. The study materials were analytically pure silver nitrate (AgNO_3_), sodium borohydride (NaBH_4_), and sodium hydroxide (NaOH) received from Merck, Germany. Nitric acid (HNO₃) (~ 65% conc.) was bought from Merck, Darmstadt, Germany. A 10 kDa molecular weight dialysis tube was obtained from Spectra Por 131270 T Biotech-Grade Dialysis Tubing (USA). The cotton textile fabric (plain weave) was 100% bleached, with approximately 150 GSM (Grams per square meter), collected from a local textile factory in Bangladesh.

### Methods

#### Silk degumming

The degumming process for SF was investigated using a method referred to as the Degumming Recipe. In this method, a 5:2 (g/mL) ratio of SF to liquid was mixed, and 4.24 g of sodium carbonate (Na_2_CO_3_) was added to the solution. The degumming process was conducted at 55 °C for 30 min. After the glue was removed, the small pieces of cocoon were cleaned and squeezed for 20 min. The cleaned SF samples were then placed on aluminium (Al) foil and dried in a fume hood overnight until they reached a consistent weight. This method was compared to four other widely used degumming methods, such as autoclave degumming, short (30 min) alkaline boiling, long (120 min) alkaline boiling, and ultrasonication with a probe in water. After each degumming step, the remaining SF was washed with distilled water to remove any unbound sericin (SS) and dried for 24 h.

#### Dissolution of SF fiber

This study employed a combination of techniques to solubilize fibers. First, a solution consisting of three components—Ajisawa’s Reagent (prepared with calcium chloride), ethanol, and water in a 1:2:6 mol ratio was used^32^. Fibers were fragmented and added to the solution at a concentration of 1 g per 10 mL. SF was incorporated to achieve a 5 wt.% concentration. The mixture was heated at 70 °C for 4 h in a microwave oven to fully dissolve the SF. Fibroin dispersions were placed on pre-hydrated cellulose membranes post-solubilization and dialyzed in ultrapure water at 25 °C for 48 h with continuous stirring. The water was replaced every 8 h, maintaining a 1:30 volume ratio between the dispersion and water^33–36^. After dialysis, the dispersions were transferred to 50 mL Falcon tubes and centrifuged at 7000–8000 rpm for 10 min using a Centurion Pro-Analytical centrifuge to remove contaminants. The supernatant was collected and dialyzed for an additional 24 h. The final fibroin solution was stored at 4 °C for future use^37,38^.

#### Synthesis of SF nanoparticles (SFNPs)

**Fig. 1:**
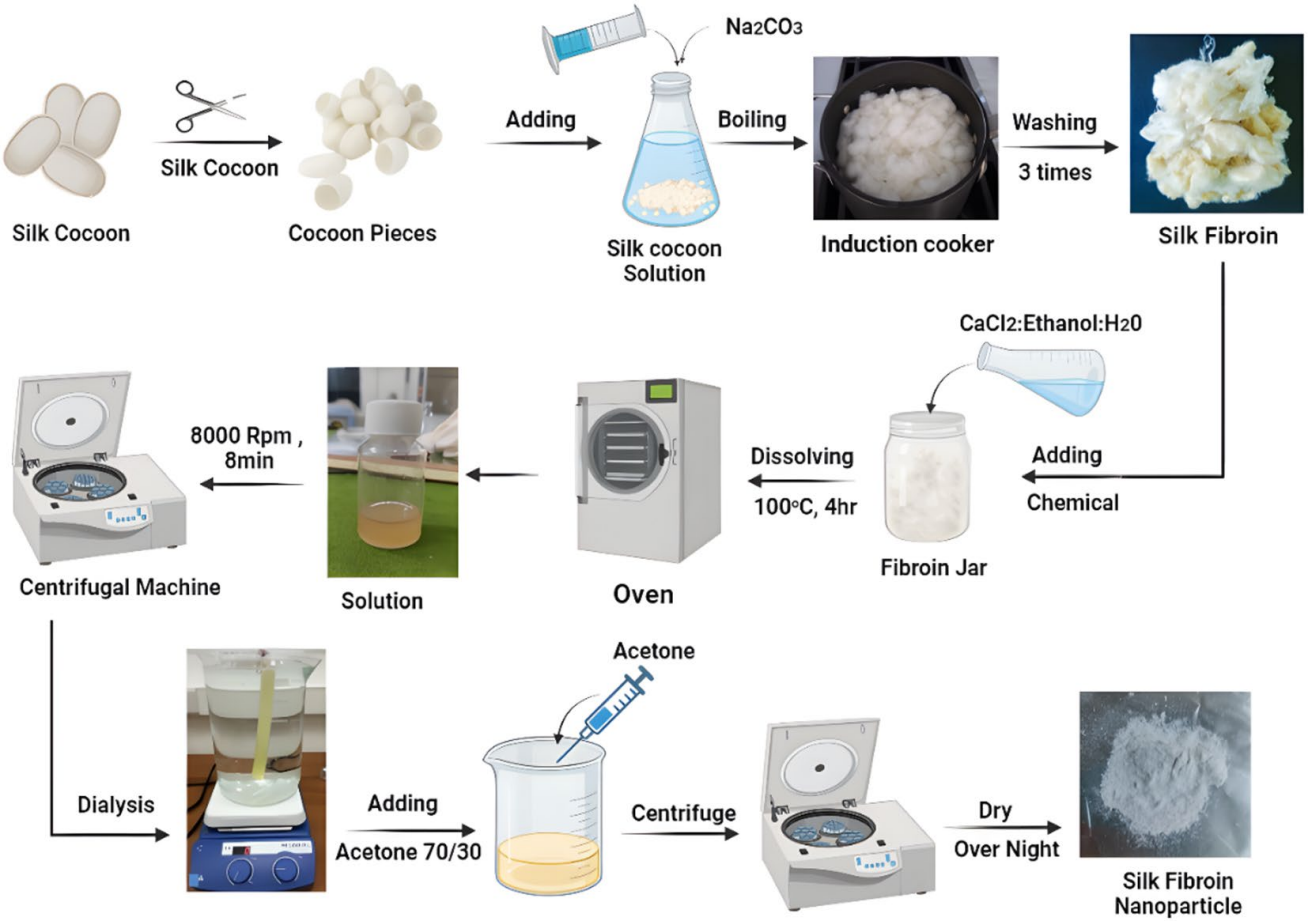
Schematic illustration of the preparation of silk fibroin nanoparticles (SFNPs).

The preparation of SFNPs followed a modified methodology based on Zhao et al.^39^. A 20 mL injector rapidly introduced an SF solution into a solvent mixture containing over 70% acetone. In a separate container, the protein solution was vigorously agitated. As illustrated in Fig. 1, this process rapidly produces milk-like silk protein particles or enzyme-encapsulating silk protein particles. Nanoparticle (NP) aggregation precipitates these water-insoluble particles. The mixture was then filtered using 0.22 μm filter paper. Centrifugation at 108,000 times gravity (× g) for 0.5 h can extract the particles from the solvent. After filtering or centrifugation, the NPs were transferred to Al-foil and placed in a fume hood for several days to allow SF nanoparticle crystallization. The crystal structure was mechanically disrupted using a mortar and pestle to obtain SFNPs powder^39^.

#### Synthesis of silver nanoparticles (AgNPs)

**Fig. 2:**
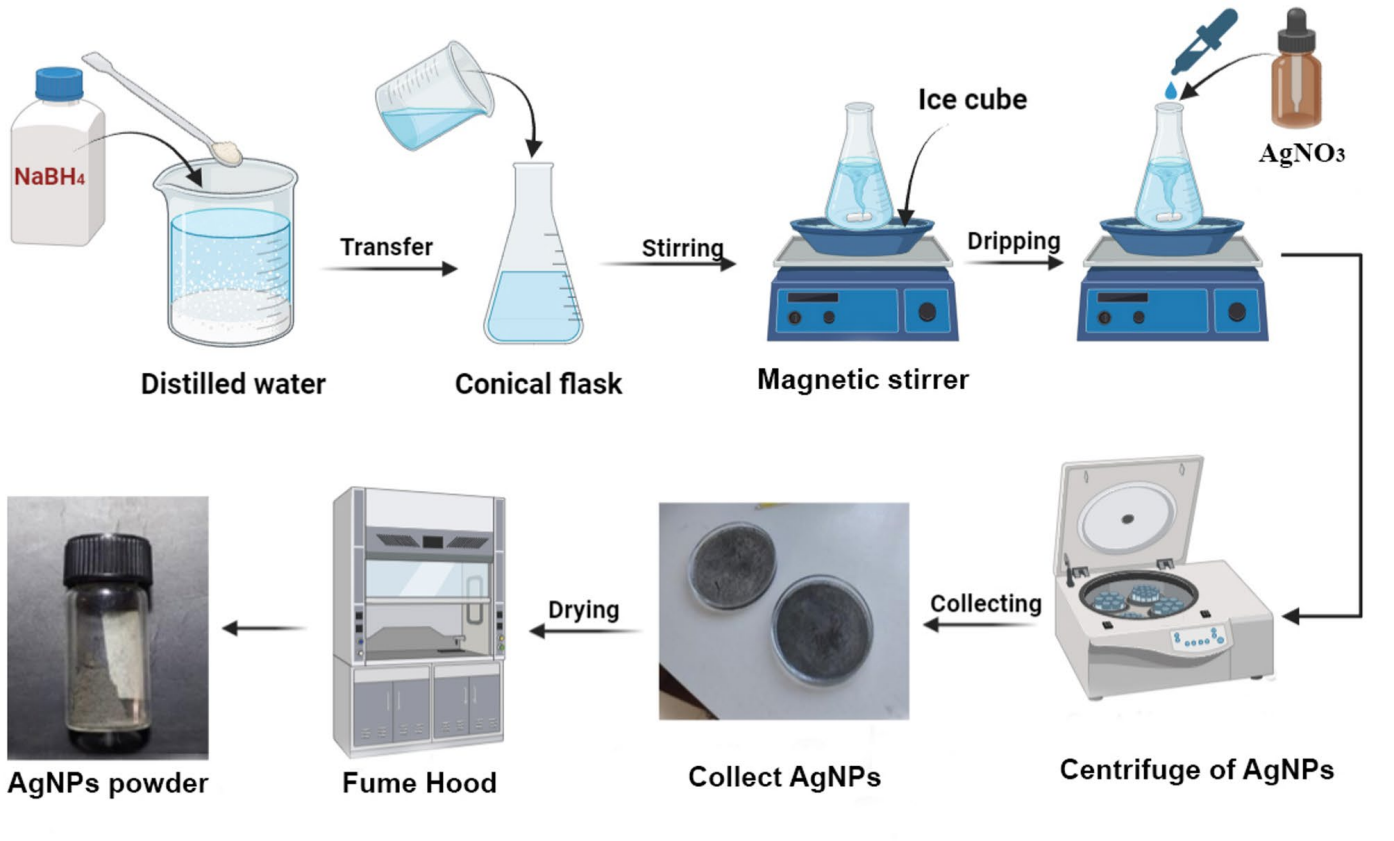
A schematic illustration of the preparation of silver nanoparticles (AgNPs).

Figure 2 illustrates the synthesis of silver nanoparticles (AgNPs). First, a silver nitrate (AgNO_3_) solution was prepared by dissolving a precise amount of AgNO_3_ in distilled water. Similarly, a sodium borohydride (NaBH_4_) solution was prepared by dissolving NaBH_4_ in distilled water. The experimental procedure involved introducing the NaBH_4_ solution into a conical flask equipped with a magnetic stirrer bar, placed within an ice bath. Subsequently, 2 mL of AgNO_3_ solution was gradually added drop by drop to the NaBH_4_ solution, ensuring a controlled process. The AgNPs solution was subsequently centrifuged to separate the nanoparticles (NPs) from any remaining unreacted materials. After centrifugation, the solution containing AgNPs was carefully collected and transferred to a fume hood to facilitate the evaporation of volatile components^40,41^.

#### Cotton fabric modification with SFNPs and AgNPs

**Fig. 3:**
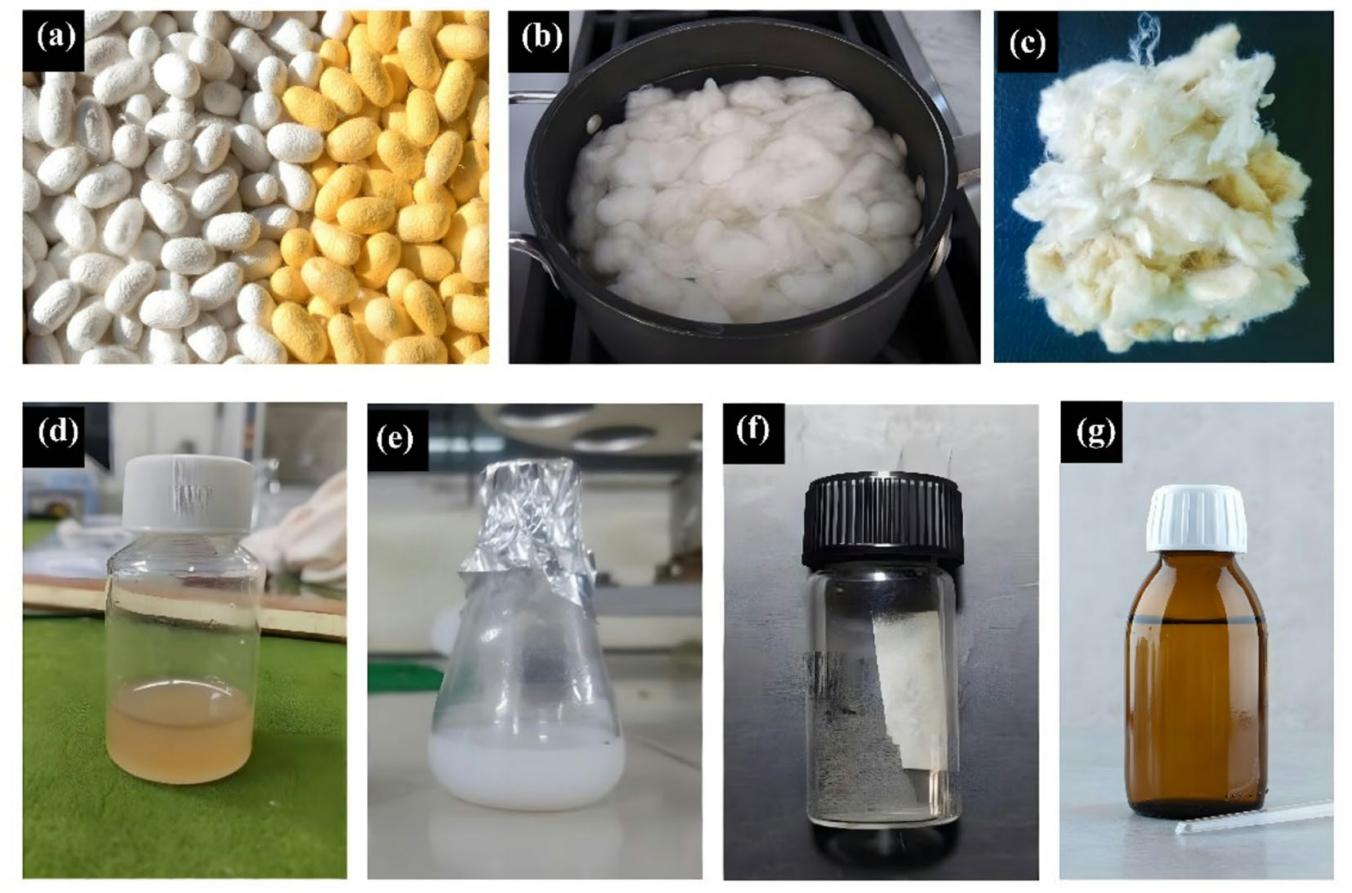
Formation of solution (**a**) Cocoons, (**b**) Silk degumming, (**c**) SF fibers, (**d**) Pure SF solution, (**e**) SFNPs solution, (**f**) AgNPs, (**g**) Colloidal SF-AgNPs.

**Fig. 4:**
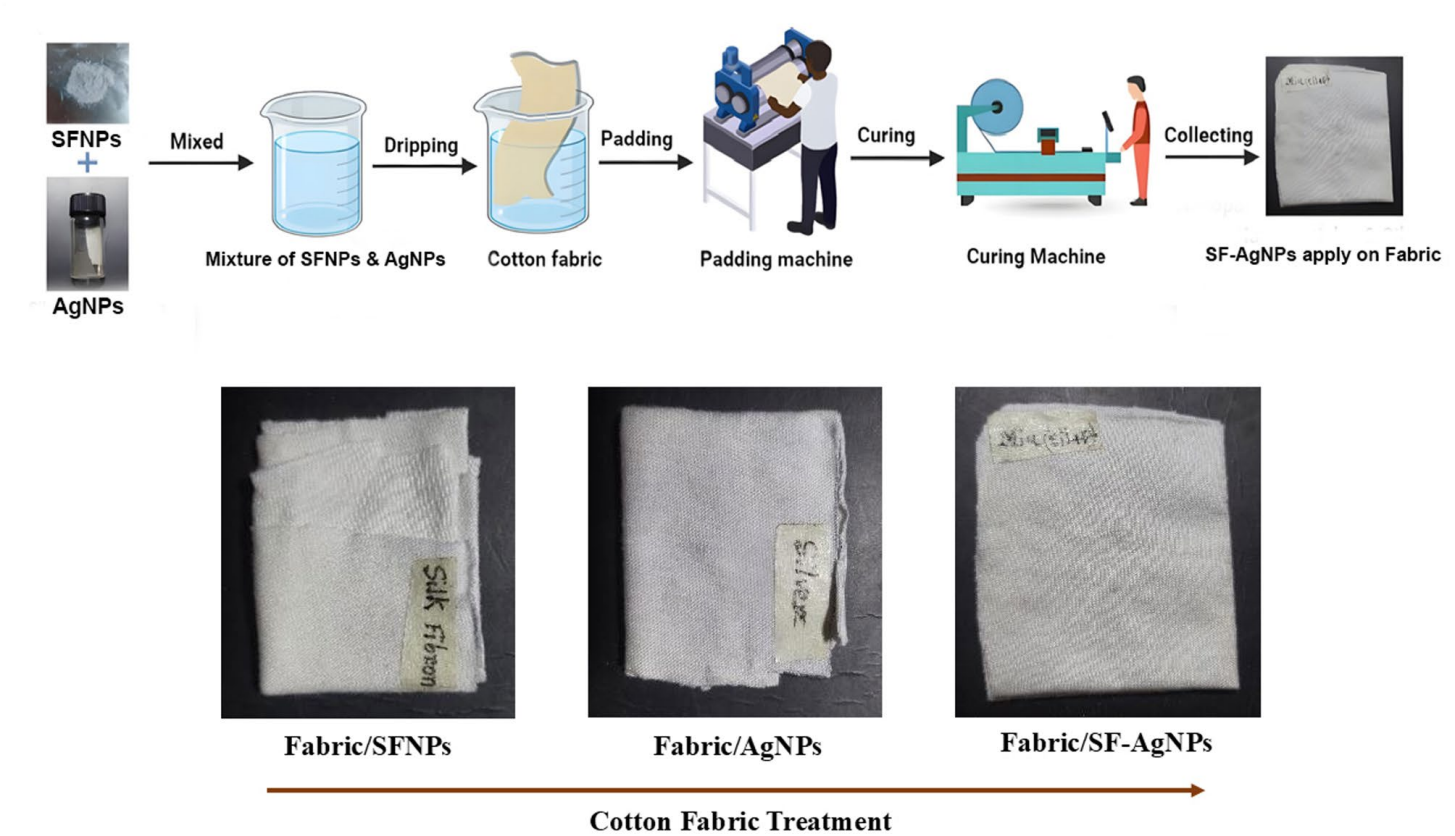
Schematic illustration of the modification process of cotton textiles with synthesized nanoparticles.

Table 1 shows the sample formulation for modifying cotton textiles, while the modification processes and required elements are illustrated in Figs. 3 and 4, respectively. First, the combined nanoparticle solution (SF-AgNPs) was prepared by adding 0.1 g of both SFNPs and AgNPs into water and stirring continuously. Once the solution was ready, the cotton fabric was immersed for 10 min, passed through a padding machine, and cured at 121 °C for 12 min. Here, S0 represents the untreated cotton samples, S1 and S2 represent the SFNPs and AgNPs-treated cotton, respectively. S3 represents the combined (SF-AgNPs) treated cotton textiles.

**Table 1:** Sample formulation for cotton textile modification.

Symbol	Treatment
S0	Untreated cotton
S1	SFNPs treated cotton
S2	AgNPs-treated cotton
S3	Cotton treated with the combined (SF-AgNPs) solution

### Characterization

#### UV–vis spectroscopy

A Shimadzu UV-1800 spectrophotometer (Shimadzu, Japan) was used to record the UV–Vis absorption spectra of SF, SFNPs, AgNPs, and SF-AgNPs within the wavelength range of 190–800 nm, covering the ultraviolet and visible regions of the electromagnetic spectrum. The measurements were conducted exclusively on nanoparticle samples.

#### X-Ray diffraction (XRD)

A Rigaku Miniflex II X-Ray diffractometer (Rigaku, Japan) was used to investigate the crystallinity index of the nanoparticles. The instrument operated at a voltage of 40 kV and a current of 40 mA, using CuKα radiation with a wavelength (λ) of 1.5406 Å. The scanning range for the samples was set from 10 to 80° in 2θ (two theta) with a scanning speed of 5°/min and a step size of 0.02°.

#### FT-IR spectral analysis

For FTIR analysis, the instrument employed was the Nicolet 5700 Fourier-transform infrared (FTIR) spectrophotometer (ThermoFisher Scientific, USA). The measurements were performed in the wavenumber range of 4000–500 cm⁻^1^, with a resolution of 4 cm⁻^1^ and 32 scans per sample to ensure a high signal-to-noise ratio.

#### Scanning electron microscopy (SEM)

Surface morphology and surface composition of samples were investigated using an AIS-2100 scanning electron microscope (SEM) equipped with energy-dispersive X-ray spectroscopy (EDS). Fabric samples were mounted on conductive carbon adhesive tabs and then sputter-coated (SC 7620 EMITECH) with a thin layer of gold before imaging. The operation voltage was adjusted to 10 kV.

#### Assessment of antibacterial activity

The qualitative antibacterial assessment of the nanoparticles (NPs) and modified cotton textiles was conducted using the disc diffusion method^42^. Gram-negative *E. coli* and Gram-positive bacteria *S. aureus* were used as the tested microorganisms. Bacterial cultures were cultivated on nutrient agar and Mueller Hinton broth (MHB) media and preserved at 4 °C. Stock solutions of SFNPs (100 mg/mL) and AgNPs (5 mg/mL) were prepared. A mixture (SFNPS + AgNPs) was made by combining 0.5 mL SFNPs solution and 0.2 mL AgNPs solution, then diluted with autoclaved distilled water. The SF-AgNPs mixture was applied to cotton fabric for antimicrobial activity testing, which was impregnated onto a sterile paper disc (5 mm diameter) and kept agar. Petri plates were incubated at 37 °C for 24 h. Before this experiment, all apparatus was sterilized via autoclaving at 121 °C for 30 min. Antimicrobial activity was measured as the diameter of the inhibition zone in mm^43,44^.

#### DPPH radical scavenging assay

The antioxidant activity of synthesized nanoparticles was determined based on their scavenging activity using the DPPH (2,2-diphenyl-2-picrylhydrazyl hydrate) assay. Different concentrations of SFNPs, AgNPs, SF-AgNPs mixture solution, and commercial sample ascorbic such as 800, 400, 200, 100, 50, 25, 12.5, and 6.25 µg/mL were individually mixed with 4 mg of DPPH on a watch glass and dissolved in 99.5% methanol of 100 ml and incubated in the dark for 30 min. Here, ascorbic acid was taken as standard. The absorbance of the samples was measured at 517 nm using a Shimadzu UV-1800 spectrophotometer^45^. Then, the antioxidant activity was calculated according to the following equation (Eq. 1)

1 $${\text{Scavenging }}\left( {\text{\% }} \right) = \frac{{{\text{Absorbance of control}} - {\text{Absorbance of test sample }}}}{{\text{Absorbance of control}}} \times 100{\text{\% }}$$

## Results and discussion

### Characterization of the synthesized nanoparticles (NPs)

#### UV–vis spectroscopy

**Fig. 5:**
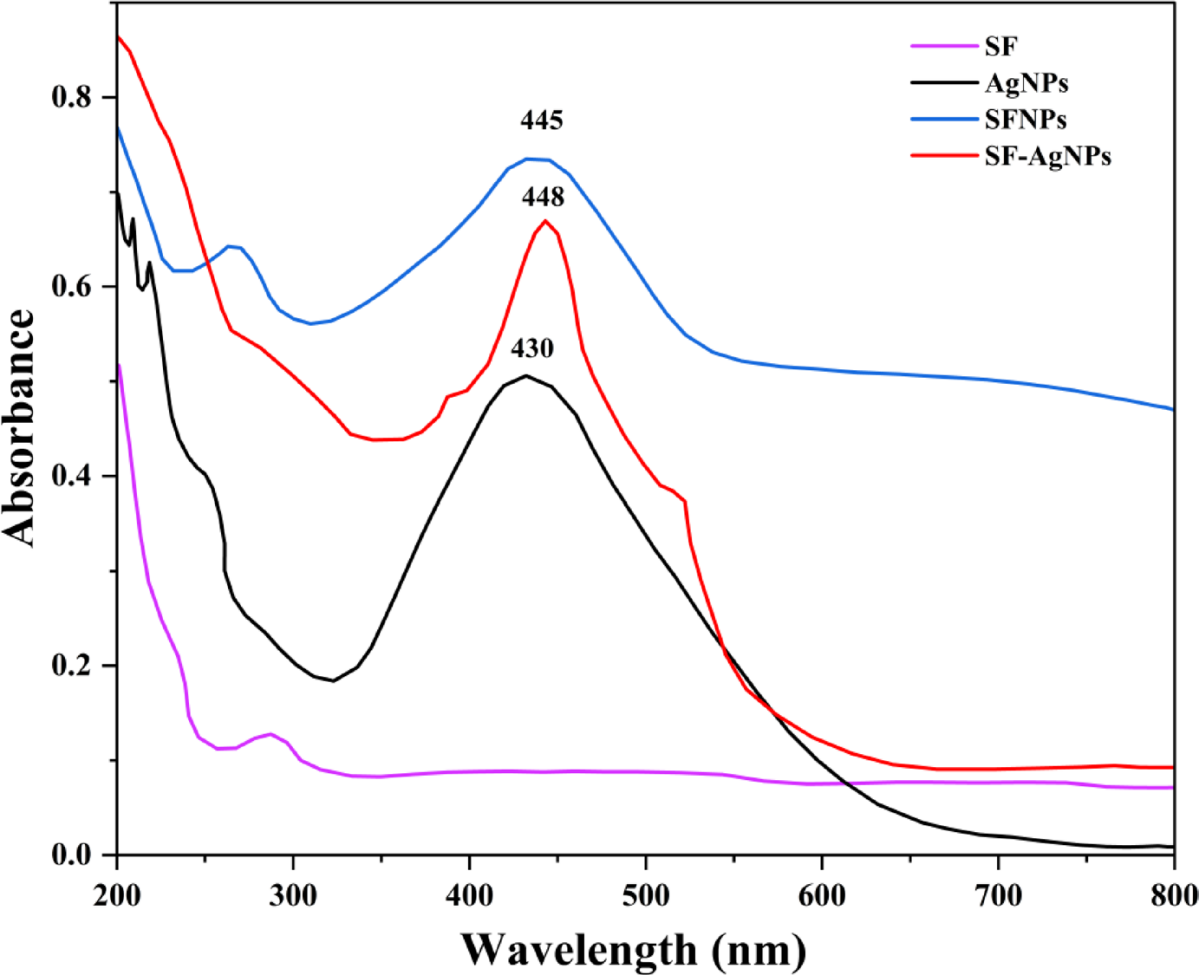
UV–Vis spectra of (**a**) SF, (**b**) AgNPs (**c**) SFNPs, (**d**) SF-AgNPs.

Figure 5 exhibits the UV–Vis absorption spectrum of an aqueous solution of pure silk fibroin (SF), which displays a strong absorption band at 276 nm. This band is primarily attributed to the π → π* transition of the tyrosine residue in the SF chain. After the reaction, the characteristic peak of tyrosine at 276 nm significantly red-shifted to 288 nm. This shift may indicate electron transfer from tyrosine to metal ions, altering the electron density in the tyrosine moiety during the reduction process^46–49^. Metal NPs show strong and vibrant colors due to their electrons` response to light (surface plasmon resonance or SPR)^50^.

Figure 5b shows the absorption spectrum for AgNPs, displaying a stable and symmetrical color at 430 nm in the visible spectrum. The symmetry suggests that the NPs have a spherical shape, while the longer tail in the red region indicates a variety of sizes^28^. Figure 5c illustrates the UV absorption spectrum of regenerated liquid silk, exhibiting two distinct peaks (a main peak and a minor peak) attributed to tyrosine and tryptophan in SF (see Fig. 5a)^51^. Upon transforming aqueous silk fibroin (SF) into NPs, the UV–vis spectrum demonstrated successful loading at wavelength at 265 nm and 445 nm into the fibroin NPs, significantly reducing the lowest UV absorption value. This transformation significantly enhanced UV absorption in the range of 200–400 nm, thereby improving the UV-shielding (inhibition) properties of the SFNPs, as also reported by Li et al.^52^. For SF-AgNPs (see Fig. 5d), three peaks are demonstrated: the first at λ_max_ = 381 nm, the second at λ_max_ = 448 nm, and the third at λ_max_ = 515 nm. The first LSPR (Localized Surface Plasmon Resonance) band at λ_max_ = 381 nm indicates the presence of triangular particles^48^. While spherical NPs typically show a single absorption band, anisotropic NPs can exhibit multiple absorption bands depending on their shape. This study demonstrates multiple LSPR bands, as reported by El-Dessouky et al.^53^ and Sangappa et al.^48^.

#### XRD analysis

**Fig. 6:**
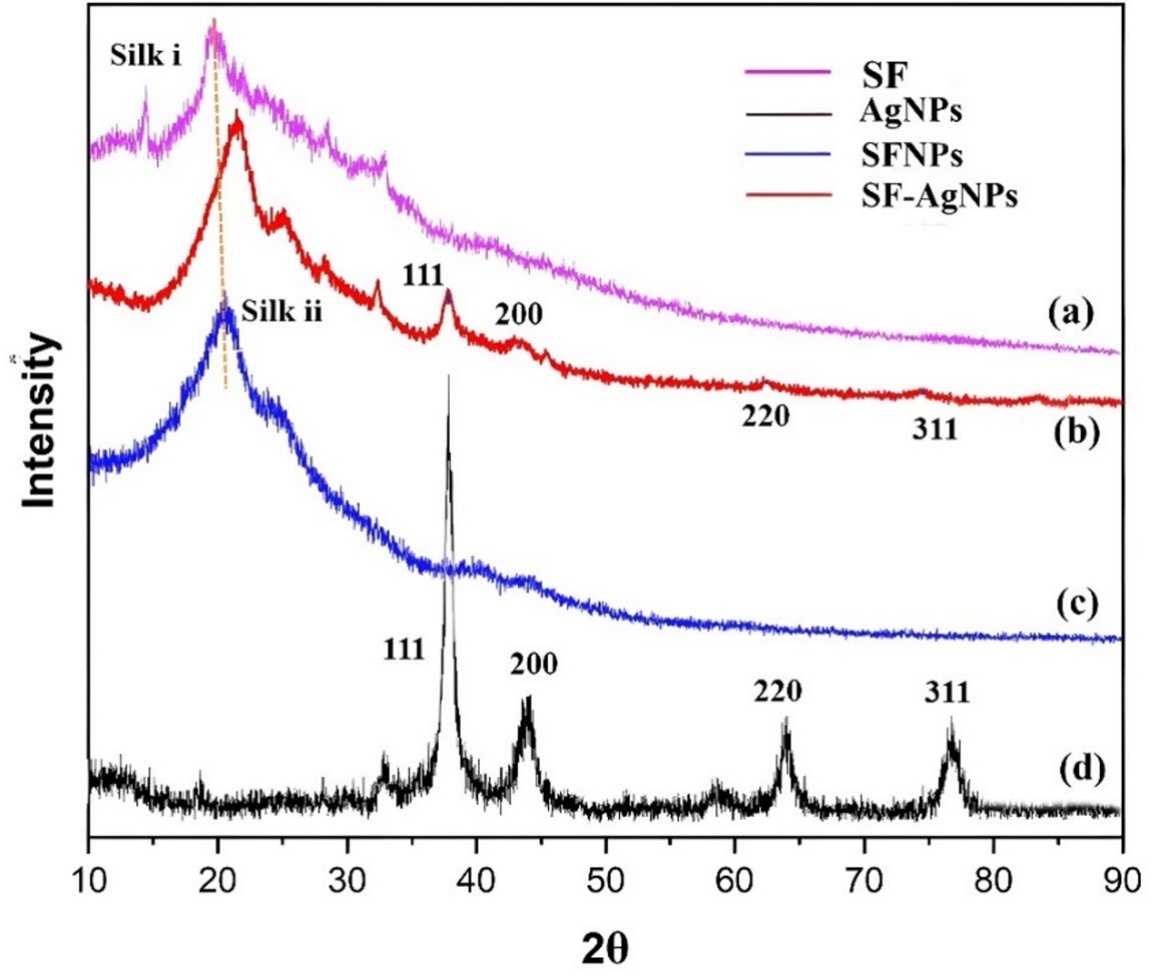
XRD patterns of (**a**) Pure SF, (**b**) SF-AgNPs, (**c**) SFNPs, (**d**) AgNPs.

Figure 6 demonstrates the XRD patterns of pure SF, AgNPs, SFNPs, and the combined solution of SF-AgNPs. For the XRD experiment, the dried powder form of these materials was employed^54^. In the case of pure SF, two distinct peaks at 2θ = 19.72° are exhibited (see Fig. 6a), corresponding to the crystalline structure of SF. The notable diffraction peaks observed at 2θ = 20.22º, 38.22º, 44.45º, and 77.32º confirm the presence of a face-centered cubic (FCC) crystalline structure of SF-AgNPs (see Fig. 6b), with corresponding crystal planes of (111), (200), (220), and (311). Additionally, a broad peak at 19.75º confirms the presence of SF in the colloidal sample, as supported by the literature^40,55–57^. The primary diffraction peaks of SFNPs (see Fig. 6c) are found in the 10–40° X-ray diffractograms, with sharp peaks at 2θ = 20.36°, indicating the conformation of SF, and at 2θ = 24.16°, representing the structure of SFNPs derived from B. mori protein^58^. The characteristic peaks of AgNPs observed in Fig. 6d at scattering angles (2θ) around 38.24°, 44.16°, 64.66°, and 77.49° can be associated with the (111), (200), (220), and (311) planes of silver (Ag), compared with the standard powder diffraction card from JCPDS, silver file no. 00–004-0783^59^. The XRD analysis confirms that the resulting particles possess a face-centered cubic (FCC) structure characteristic of metallic AgNPs. The average grain size of the AgNPs formed during the bioreduction process was calculated using the Debye–Scherrer formula: L = (kλ)/(βcosθ), where L represents the average grain size, k is the Scherrer constant, λ is the X-ray wavelength, β is the full width at half maximum (FWHM) of the measured reflection, and θ is the diffraction angle^60–62^.

#### FTIR analysis

**Fig. 7:**
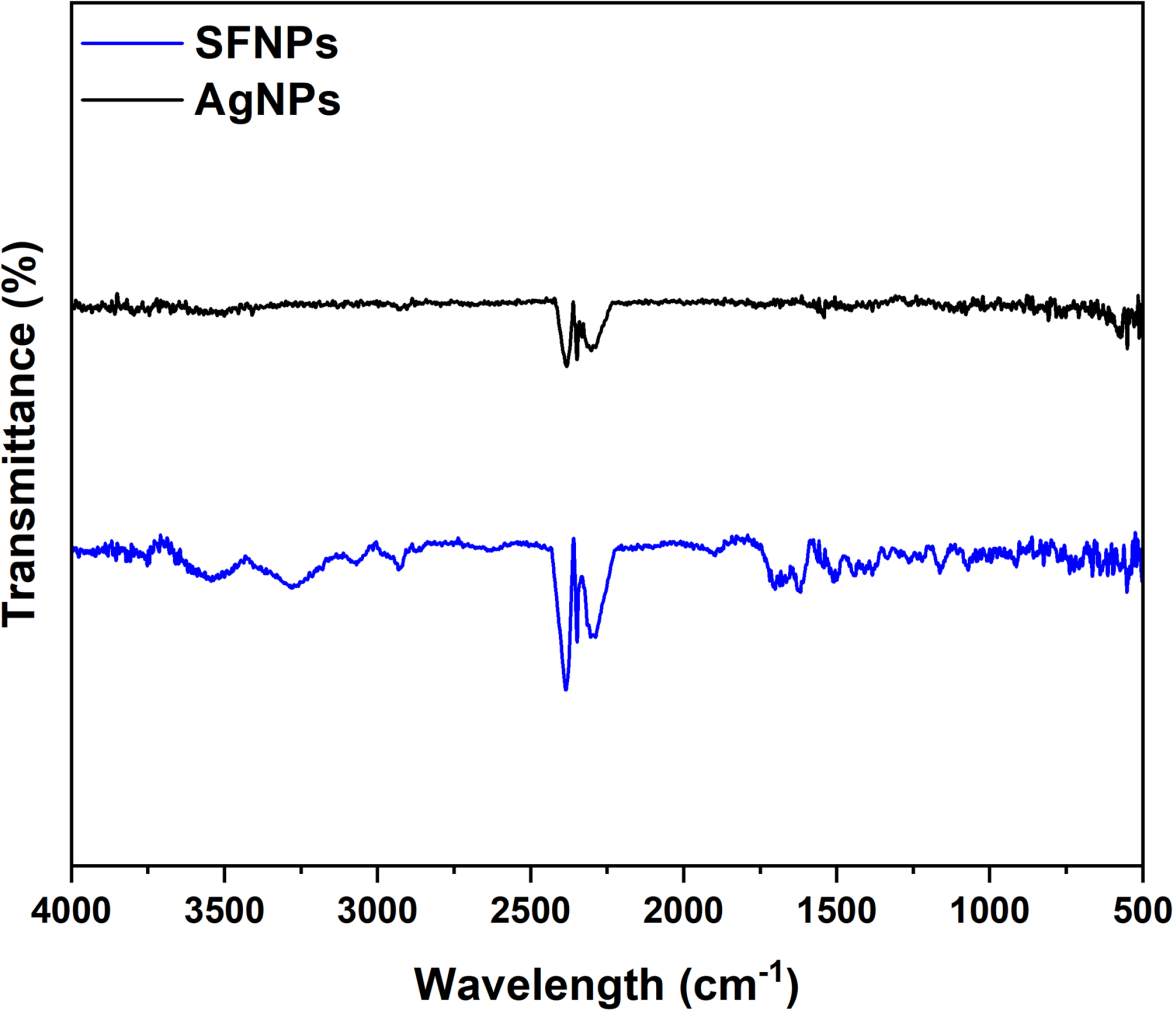
FTIR analysis of synthesized nanoparticles (**a**) SFNPs, and (**b**) AgNPs.

Figure 7 illustrates the FTIR spectra of the synthesized nanoparticles, including SFNPs and AgNPs. The amide I peak observed in the 1680–1400 cm^−1^ range corresponds to the carbonyl (C = O) stretching vibration of carboxylate ions coupled to the amide linkage^63^. The amide II peak at 1544.17 cm^−1^ represents the C–H stretching vibration and N–H bending in the amide linkage, providing valuable information about conformational changes in the silk II structure^64^ observed in Fig. 7a. Additionally, peaks observed in the 1480–1335 cm^−1^ range are attributed to the symmetric stretching vibrations of carboxylate and methyl alanine groups in the protein. The disappearance of the peak at 1060 cm^−1^, originating from the phenolic groups of the tyrosine molecule, indicates that phenolic residues are likely to have formed chelation complexes with ions present in the solution. Previous studies by Dong et al.^65^ have demonstrated that SF proteins can bind to metal ions through free amine groups or cysteine residues in the protein structure.

FTIR spectrum reveals two bands at 1577 and 1329 cm^−1^ corresponding to the bending vibrations of the amide I and O–H groups of the proteins, respectively,^39^ as shown in Fig. 7b. The absorbance band at 1021 cm^−1^ is due to the stretching vibration corresponding to the C–N group, while the bands at 837, 729 and 689 cm^−1^ are due to bending vibrations. The absorbance band at 2929 cm^−1^ is associated with the C–H cm^−1^ stretching vibration. The bending vibration at 1329 cm^−1^ and the broad absorption band at 3000–3500 cm^−1^ likely arise from the OH group of polyols such as hydroxyflavones and catechins^40^. Similar findings of FTIR peaks were obtained by Mallikarjuna et al.^66^ and Rajendrachari et al.^67^.

### Characterization of the modified cotton textiles

#### FTIR

**Fig. 8:**
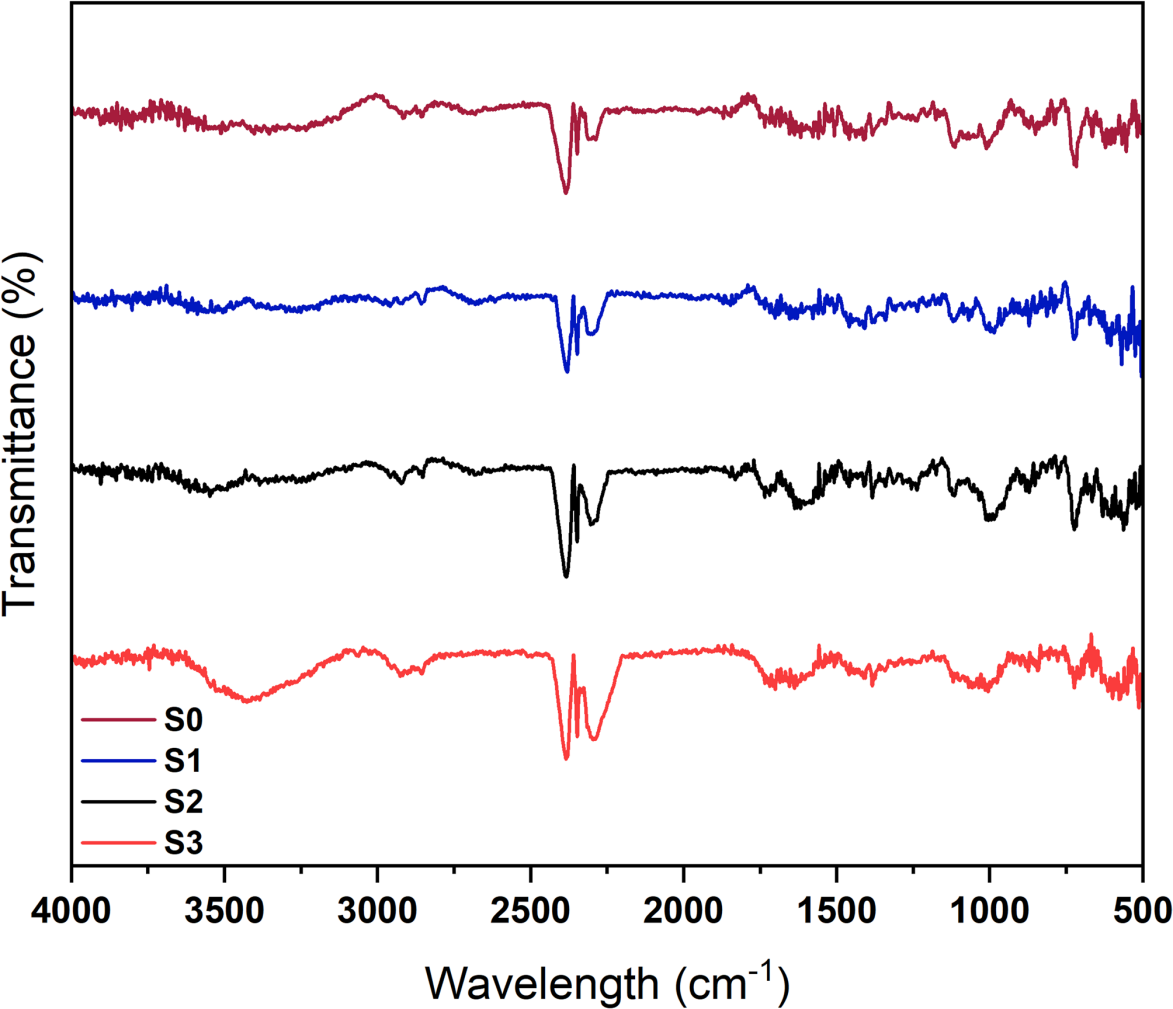
FTIR analysis of (**a**) Untreated cotton fabric (S0), (**b**) Treated fabric with SFNPs (S1), (**c**) Treated fabric with AgNPs (S2), and (**d**) Treated fabric with a mixture of SF-AgNPs (S3).

The FTIR analysis of the four samples demonstrates characteristic cellulose peaks, confirming the successful treatment of the cotton fabric with NPs, as shown in Fig. 8. The FTIR spectra of untreated cotton textile (S0) exhibit characteristic cellulose peaks in the range of 1000–1200 cm⁻^1^^68^, along with a broad peak at 3500–3100 cm⁻^1^ corresponding to hydrogen-bonded OH stretching, and a peak at 3000–2800 cm⁻^1^ for C–H stretching. Additionally, a peak around 1647 cm⁻^1^ is attributed to adsorbed water molecules. These peaks are consistent with the chemical structure of cellulose and serve as a reference for the untreated cotton compound^6,69^.

Peak intensity changes were detected in the 3000 cm⁻^1^ and 3500 cm⁻^1^ regions in the spectra of the treated samples (S1, S2, and S3), related to the cellulose hydroxyl group. The untreated cotton textile (S0) exhibited stronger peak intensities, indicating the existence of –OH groups prior to nanoparticle application. The peak intensity decreased after applying SFNPs (S1), AgNPs (S2), and the mixture of SF-AgNPs (S3). This reduction in peak intensity signifies the interaction of cellulose hydroxyl groups with the nanoparticles, likely through hydrogen bonding and coordination of surface silver species on AgNPs^70^.

The changes in the FTIR spectra validate the successful nanoparticle loading onto the cotton textiles. The existence of characteristic cellulose peaks in all samples and the reduction in intensities of the surface hydroxyl groups in the treated samples clearly prove that the application of the nanoparticles was successful. These findings align with the interactions between SF and AgNPs and the changes in functional groups on cotton textiles after applying SFNPs or AgNPs. Similar observations were reported in the FTIR spectra by Wang et al.^71^, Shivananda et al.^49^, and Chung et al.^69^.

#### SEM–EDS analysis

**Fig. 9:**
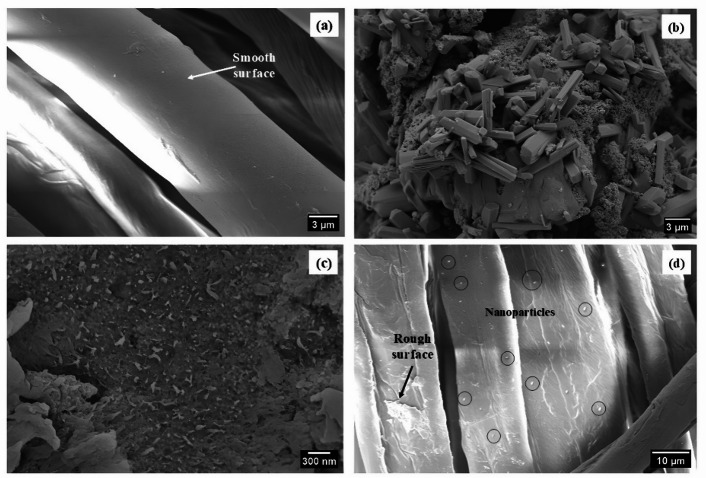
SEM images of (**a**) S0, (**b**) SFNPs, (**c**) AgNPs, (**d**) S3.

The scanning electron microscopic (SEM) analysis of the untreated cotton textile (S0), SFNPs, AgNPs, and Cotton treated with combined (SF-AgNPs) solution (S3) provides strong evidence of successful implementation and morphological changes induced by NPs treatments, as presented in Fig. 9. The SEM microimages of the untreated cotton fabric (S0) in Fig. 9a indicate a smooth and even surface at varying magnifications, establishing a baseline of the absence of any NPs or surface changes. In contrast, the NPs-treated cotton textiles exhibit a distinctly rough and irregular surface morphology as demonstrated in Fig. 9d. The deposition of protein-based nanoparticles (SFNPs) and silver nanoparticles (AgNPs) on the cotton fabric is clearly visible, with the NPs evenly distributed on the surface^33^. The fabric with a blend of SFNPs and AgNPs shows even higher surface roughness, indicating the synergistic effect between the two types of NPs. Comparative analysis with the untreated textiles (S0) confirms that the increased surface roughness in the treated samples is entirely due to the NPs^72,73^.

**Fig. 10:**
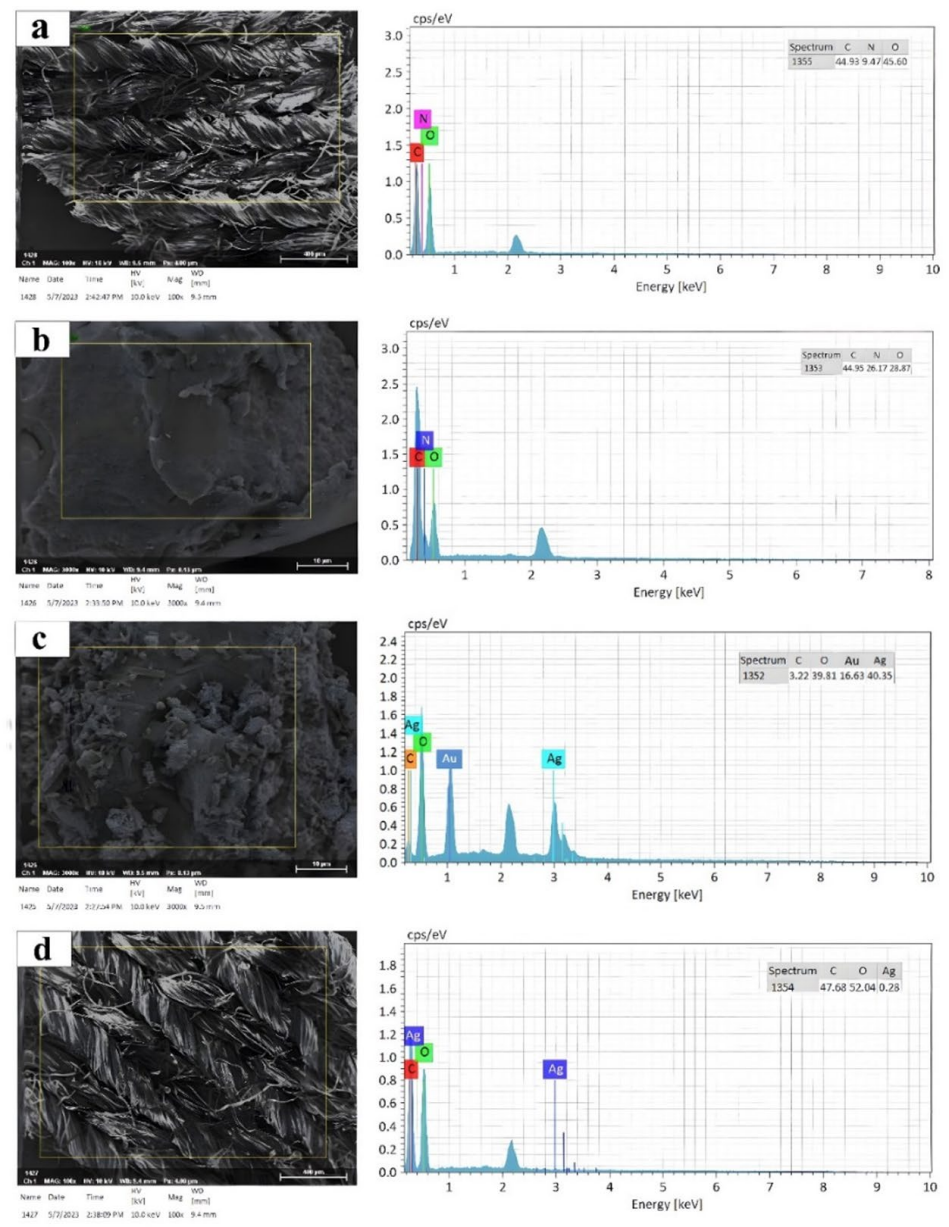
Energy-dispersive X-ray spectroscopy (EDS) spectra: (**a**) S0, (**b**) SFNPs, (**c**) AgNPs, and (**d**) S3.

Figure 10 shows the EDS spectra for the elemental composition analysis of S0, SFNPs, AgNPs, and S3. The EDS analysis results, along with the corresponding SEM images, provided valuable information about the composition and morphology of the samples. The peaks in the spectrum were attributed to carbon (C) and oxygen (O), which are typical elements in cotton fibers^74^.

In some cases, a strong peak of gold (Au) at approximately 2.0 keV was detected, indicating the presence of a gold coating on the fabric for SEM imaging purposes^75^. Similarly, the EDS analysis of SFNPs (see Fig. 10b) showcased peaks corresponding to C, nitrogen (N), and O. Combined with SEM images, the results in Fig. 9b indicate the successful formation of SFNPs. For AgNPs, a new peak emerged in the EDS spectrum at around 3.0 keV, corresponding to the silver (Ag) signal, as shown in Fig. 10c. Comparing the SEM images confirms the successful formation of AgNPs. For the combined modification of cotton textiles with SF-AgNPs (for S3 sample), as demonstrated in Fig. 10d, it is evident that the NPs of SF and Ag are deposited on the cotton textiles, as indicated by the corresponding peaks for C, O, and Ag elements^76^. The obtained elemental peaks in the EDS spectra, supported by SEM images, confirm the accurate identification and successful formation of the NPs on the cotton textile surfaces.

#### Antimicrobial activity

The antibacterial activity of SFNPs, AgNPs, SF-AgNPs, and treated cotton with combined (SF-AgNPs) solution was evaluated using the disc diffusion assay. Two pathogenic bacteria, *S. aureus* and *E. coli*, were selected for the antibacterial tests^77^. Positive controls, including Doxycycline (30 µg/disc) and Erythromycin (15 µg/disc), were used for comparison. The results are summarized in Table 2.

**Table 2:** Antimicrobial activity zone of inhibition results.

Bacteria	Zone of inhibition (diameter in mm)					
	SFNPs	AgNPs	SF-AgNPs mixture	S3 sample	Erythromycin	Doxycycline
*E. coli*	–	8.5 ± 0.5	10 ± 0	8 ± 0	Not used	Resistant
*S. aureus*	–	9 ± 0	9.5 ± 0.5	7 ± 0	Resistant	Not used

**Fig. 11:**
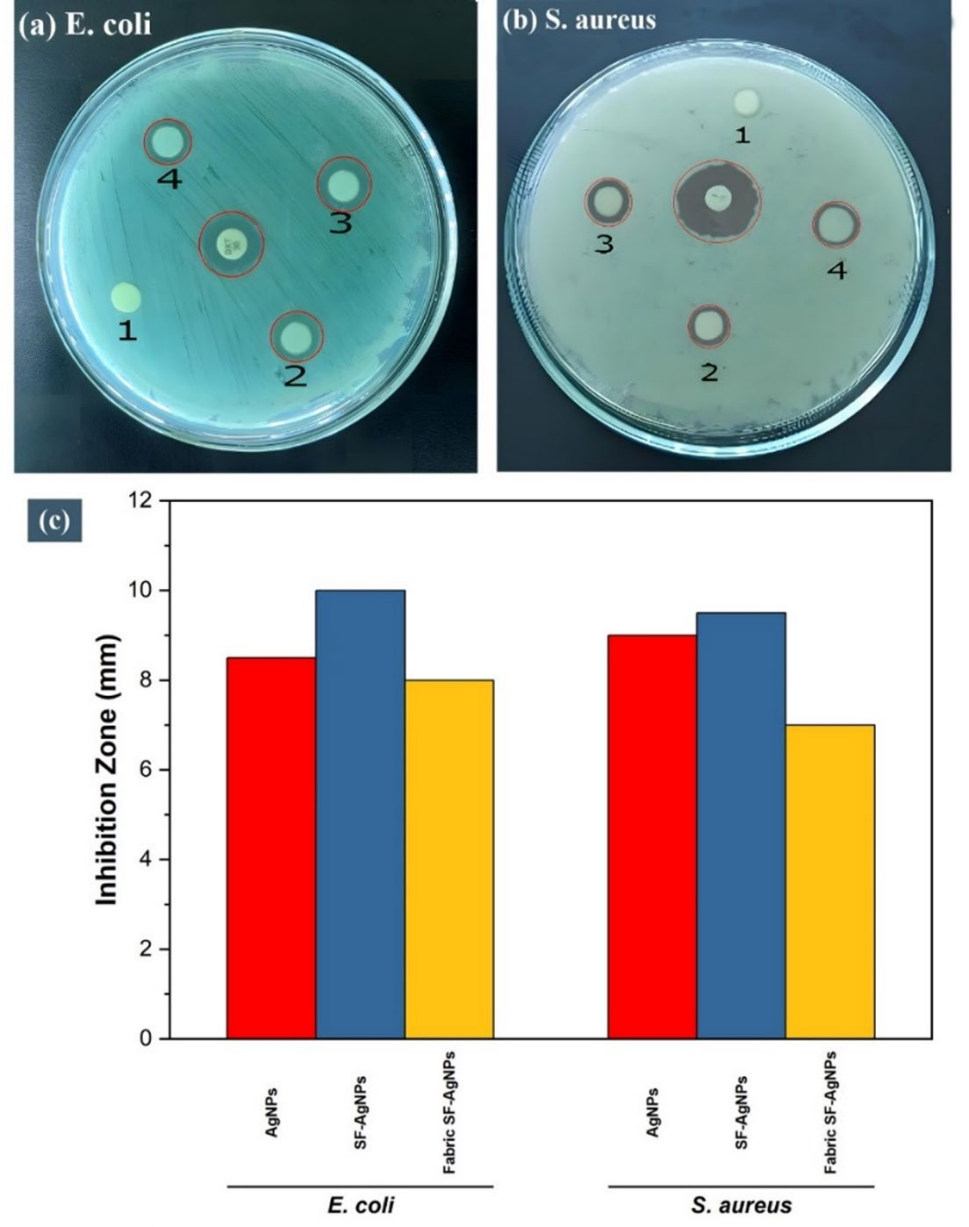
Antibacterial activity of (1) SF, (2) AgNPs, (3) SF-AgNPs (4) S3 sample against (**a**) *E. coli* and (**b**) *S. aureus*, (**c**) Inhibition zone (mm).

The AgNPs, SF-AgNPs solution, and treated textiles with SF-AgNPs mixture exhibited significant antibacterial activity against both *S. aureus* and *E. coli, as* depicted in Fig. 11. However, SFNPs alone showed no antibacterial activity against *S. aureus* and *E. coli*. The zone of inhibition, indicating the diameter of growth inhibition, was measured for each sample.

The AgNPs exhibited a zone of inhibition of 9 mm against *S. aureus, while* the SF-AgNPs solution exhibited a slightly higher zone of 9.5 mm. The S3 sample with SF-AgNPs exhibited a zone of inhibition of 7 mm against *S. aureus*. Against *E. coli*, the AgNPs exhibited a zone of inhibition of 8.5 mm, and the combined SF-AgNPs solution exhibited a higher zone of 10 mm. The S3 sample exhibited a zone of inhibition of 8 mm against *E. coli, which* was stronger than that against *S. aureus*. The varying diameters of the zones can be attributed to differences in bacterial cell wall structure and the strains’ sensitivities to Ag^+^ ions^78^. The antibacterial action of AgNPs and SF-AgNPs can be attributed to the discharge of Ag⁺ ions that bind to bacterial cell membranes and intracellular contents. These Ag^+^ ions disrupt the integrity of bacterial cell walls and cell membranes, leading to leakage and cell death^79^. The addition of silk fibroin (SF) in SF-AgNPs may enhance the stability and controlled delivery of the Ag^+^ ions, resulting in marginally higher zones of inhibition compared to AgNPs alone, as reported in the literature^80,81^. SFNPs alone showed no antibacterial action on the tested bacterial strains, highlighting the crucial role of Ag^+^ ions in achieving antibacterial effects.

#### Antioxidant activity

**Fig. 12:**
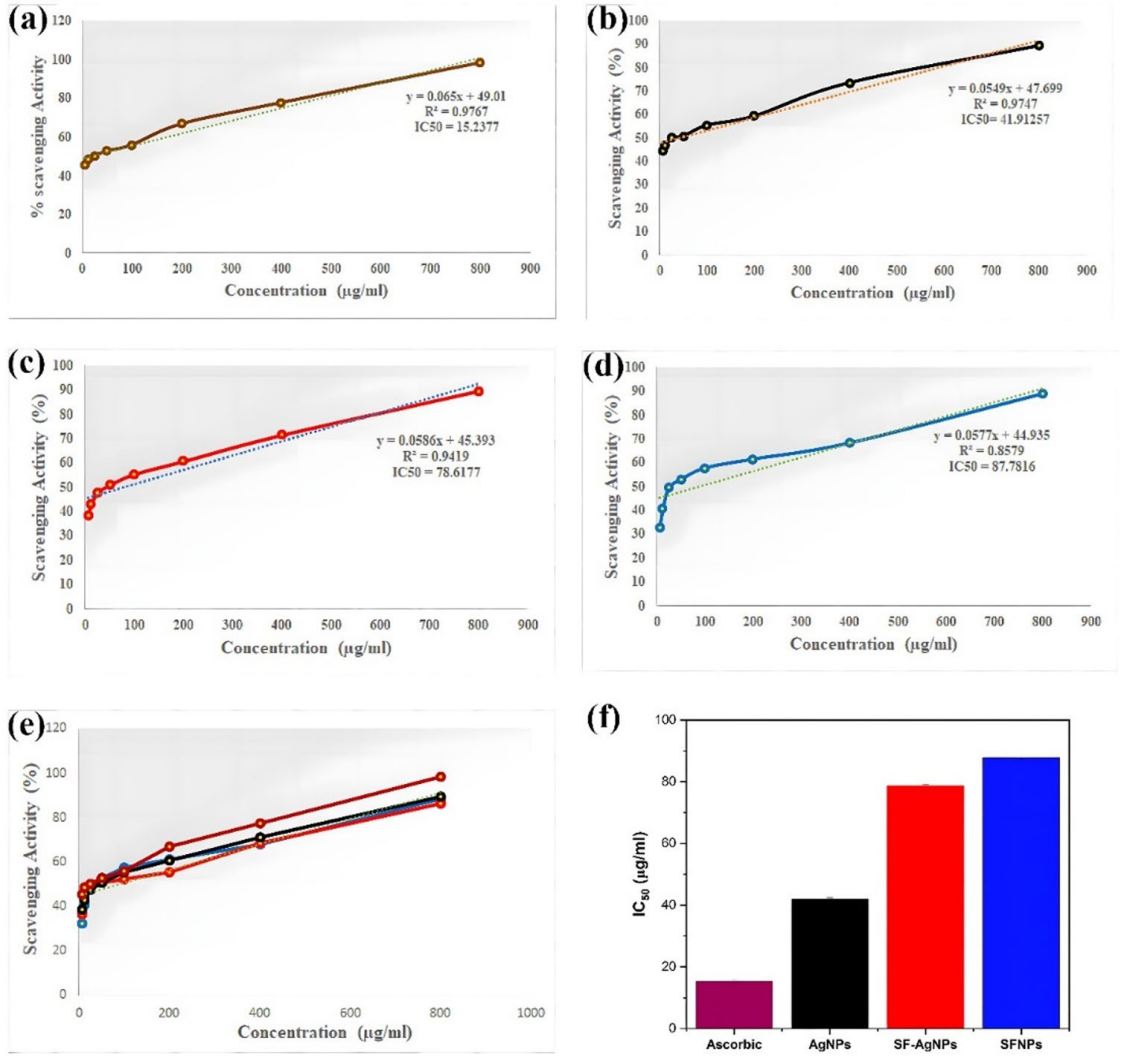
DPPH free radical scavenging activity of (**a**) Ascorbic acid, (**b**) AgNPs, (**c**) SF-AgNPs mixture solution, (**d**) SFNPs, (**e**) comparison of all samples and ascorbic acid, (**f**) IC_50_ value of samples and ascorbic acid.

Antioxidants are molecules that prevent the oxidation of other molecules, which can lead to cell damage by generating free radicals. One way to measure the antioxidant capacity of compounds is by evaluating their ability to scavenge the DPPH (2,2-diphenyl-1-picrylhydrazyl) free radical. DPPH is a stable free radical with an unpaired electron, causing it to exhibit absorption at 517 nm^82^. When an antioxidant interacts with DPPH, it donates a hydrogen atom or electron, changing the color from purple to yellow^83^. The current study compares the free radical scavenger activities of different solutions, such as SFNPs, AgNPs, and blended SF-AgNPs, with commercially purchased ascorbic acid (see Fig. 12).

The results showed that the antioxidant activity in all the solutions, including the synthesized NPs and ascorbic acid, was dose-dependent. As the concentration of the samples increased, so did the antioxidant effect, as evidenced by the rise in DPPH radical scavenging percentage^84^. The dose-dependent relationship is a characteristic feature of antioxidant activity, indicating that the compounds neutralize free radicals in a concentration-dependent manner^85^.

The IC50 value, which is the concentration of an antioxidant required to scavenge 50% DPPH free radicals, was calculated for each solution using data from Fig. 12f. A low IC50 value indicates higher antioxidant activity^86^. The derived IC50 values for the solutions were: Ascorbic Acid (15.2377 µg/ml), AgNPs (41.9125 µg/ml), SFNPs (87.7816 µg/ml), and the combined (SF-AgNPs) solution (78.6177 µg/ml). Interestingly, recent research by Vilas et al.^87^, found that AgNPs possess the maximum DPPH radical scavenging activity among the tested compounds.

However, in the present work, the combined (SF-AgNPs) solution exhibits much higher DPPH radical scavenging activity than SFNPs alone, indicating the synergistic interaction between SFNPs and AgNPs in enhancing their antioxidant activities.

## Conclusions

This work successfully synthesized silk fibroin nanoparticles (SFNPs) and silver nanoparticles (AgNPs) and employed them to functionalize cellulosic textiles, enhancing their properties against microorganisms for biomedical applications. Analytical tests, including XRD, FTIR, and UV–Vis, confirmed the existence of SFNPs and AgNPs. The XRD test verified the formation of AgNPs with a face-centered cubic (FCC) crystalline structure, while SEM analysis exhibited significant surface roughness increment, confirming the successful integration of SFNPs and AgNPs onto the cellulosic textiles. The nano-functionalized textile fabrics demonstrated good antimicrobial durability against *S. aureus* (9.5 mm zone of inhibition) and *E. coli* (10 mm zone of inhibition) and exhibited better antioxidant capacity with an IC50 value of 78.62 µg/mL in the SF-AgNPs formulation. Furthermore, the antioxidant capacity of the SF-AgNPs mixture was significantly higher than that of SFNPs alone, indicating a synergistic effect. These findings highlight the potential application of modified cellulosic textiles in biomedical fields. Future work can focus on environmental sustainability analysis, cost-effectiveness, and circularity of the overall process from nanoparticle synthesis to final application in modified textiles to ensure industrial applicability and market acceptance.

## Data Availability

All data generated or analyzed during this study are included in this published article.
